# Clinical Improvements by Telemedicine Interventions Managing Type 1 and Type 2 Diabetes: Systematic Meta-review

**DOI:** 10.2196/23244

**Published:** 2021-02-19

**Authors:** Claudia Eberle, Stefanie Stichling

**Affiliations:** 1 Medicine with specialization in Internal Medicine and General Medicine Hochschule Fulda - University of Applied Sciences Fulda Germany

**Keywords:** type 1 diabetes, type 2 diabetes, eHealth, telemedicine, disease management, systematic review, mobile phone

## Abstract

**Background:**

Diabetes mellitus (DM) is one of the world’s greatest health threats with rising prevalence. Global digitalization leads to new digital approaches in diabetes management, such as telemedical interventions. Telemedicine, which is the use of information and communication technologies, may provide medical services over spatial distances to improve clinical patient outcomes by increasing access to diabetes care and medical information.

**Objective:**

This study aims to examine whether telemedical interventions effectively improve diabetes control using studies that pooled patients with type 1 diabetes mellitus (T1DM) and type 2 diabetes mellitus (T2DM), and whether the benefits are greater in patients diagnosed with T2DM than in those diagnosed with T1DM. We analyzed the primary outcome glycated hemoglobin A_1c_ (HbA_1c_) and the secondary outcomes fasting blood glucose (FBG), blood pressure (BP), body weight, BMI, quality of life (QoL), cost, and time saving.

**Methods:**

Publications were systematically identified by searching Cochrane Library, MEDLINE via PubMed, Web of Science Core Collection, Embase, and CINAHL databases for studies published between January 2008 and April 2020, considering systematic reviews (SRs), meta-analyses (MAs), randomized controlled trials (RCTs), and clinical trials (CTs). Study quality was assessed using the *A Measurement Tool to Assess Systematic Reviews*, *Effective Public Health Practice Project*, and *National Institute for Health and Care Excellence* qualitative checklist. We organized the trials by communication technologies in *real-time video or audio interventions*, *asynchronous interventions*, and *combined interventions* (synchronous and asynchronous communication).

**Results:**

From 1116 unique citations, we identified 31 eligible studies (n=15 high, n=14 moderate, n=1 weak, and n=1 critically low quality). We selected 21 SRs and MAs, 8 RCTs, 1 non-RCT, and 1 qualitative study. Of the 10 trials, 3 were categorized as *real-time video*, 1 as *real-time video and audio*, 4 as *asynchronous*, and 2 as *combined* intervention. Significant decline in HbA_1c_ levels based on pooled T1DM and T2DM patients data ranged from −0.22% weighted mean difference (WMD; 95% CI −0.28 to −0.15; *P*<.001) to −0.64% mean difference (95% CI −1.01 to −0.26; *P*<.001). The intervention effect on lowering HbA_1c_ values might be significantly smaller for patients with T1DM than for patients with T2DM. Evidence on the impact on BP, body weight, FBG, cost effectiveness, and time saving was smaller compared with HbA_1c_ but indicated potential in some publications.

**Conclusions:**

Telemedical interventions might be clinically effective in improving diabetes control overall, and they might significantly improve HbA_1c_ concentrations. Patients with T2DM could benefit more than patients with T1DM regarding lowering HbA_1c_ levels. Further studies with longer duration and larger cohorts are necessary.

## Introduction

### Background

Diabetes mellitus (DM) is one of the world's greatest health threats [1]. The global prevalence of DM will increase from around 463 million (2019) to approximately 700 million in 2045, that is, by 51% [2]. DM is a chronic metabolic disorder associated with insulin resistance and hyperglycemia [1]. An increased blood sugar level can lead to long-term damage to the heart, eyes, nerves, kidneys, and blood vessels [3].

Type 1 diabetes mellitus (T1DM) is based on autoimmune beta cell destruction, leading to absolute insulin deficiency, whereas type 2 diabetes mellitus (T2DM) is based on a progressive loss of beta cell insulin secretion based on the background of insulin resistance [1]. A healthy diet, regular physical activity, medication, normal body weight, and blood glucose control are important therapy components to mitigate or delay consequences [3]. Access to affordable therapy is critical to the survival of diabetes patients [3]. Effective therapies are important for diabetes control. Patients with diabetes need to understand the disease and be actively involved in diabetes management for optimal therapeutic effects [4].

Global digitalization offers innovative digital opportunities for intensive diabetes management. Diabetes technology includes hardware, software, and technical devices that help to control the disease with regard to the therapy components mentioned [4]. Technological possibilities are constantly evolving and rapidly growing. Telemedical interventions in the context of diabetes management show the potential to effectively improve diabetes control [5]. Telemedicine, a term shaped in the 1970s, characterizes the “use of [information and communication technologies] to improve patient outcomes by increasing access to care and medical information” [6]. Telemedicine offers the following features: providing clinical support, overcoming geographical and physical limits, improving health-related outcomes, and applying information and communication technologies [6].

Telemedicine is a part of eHealth, defined as the use of information and communication technologies for health, which was developed in the 1990s [6]. Nowadays, the term *digital health* is recommended as it creates a link between digitalization, health, lifestyle, and community [7]. Telemedicine provides certain benefits, such as an improved access to health care services, an enhanced quality of health and care management, and potential cost and time efficiencies [8]. The model for assessment of telemedicine (MAST) provides a structured framework consisting of different domains for appraising the effectiveness of telemedicine interventions [9]. We have considered the following MAST domains as part of this research: (1) health problems and description of the application, (2) clinical effectiveness, (3) patient perspectives, (4) economic aspects, and (5) organizational aspects.

However, although lifestyle changes are part of both T1DM and T2DM management, the focus of T1DM is on insulin therapy and that of T2DM is on nutrition and regular exercise [10]. Telemedical diabetes management may be more effective in T2DM patients by addressing modifiable factors such as nutrition and exercise [10]. Furthermore, T1DM patients are younger, and the management of T1DM in childhood and adolescence is both a medical and psychosocial challenge, as adolescence is a very vulnerable period [11].

### Objectives

We recently examined the clinical effectiveness of telemedical interventions for diabetes therapy in T1DM and T2DM patients separately. Tendency differences in the clinical effectiveness of telemedical interventions between these two types of diabetes require further research in greater detail. After these separate analyses, that is, on the one hand, the analysis of T1DM patients and on the other hand that of T2DM patients exclusively, we want to focus on publications that include both types of diabetes. Therefore, in this systematic meta-review, we examined 2 hypotheses using studies that pooled T1DM and T2DM participants. We hypothesize that (1) telemedical interventions effectively improve diabetes control overall and (2) the benefits may be significantly greater in patients diagnosed with T2DM than in patients diagnosed with T1DM. As part of the first hypothesis, we also aimed to identify which intervention types (technologies) are particularly successful and effective. The focus of this systematic meta-review is on the communication between patients and health care professionals. As part of the second hypothesis, we focused on studies that specifically investigated differences between T1DM and T2DM patients with regard to the clinical effectiveness of telemedical interventions. In general, we concentrated on the following clinically relevant outcomes: primary outcome glycated hemoglobin A_1c_ (HbA_1c_) and secondary outcomes fasting blood glucose (FBG), blood pressure (BP), body weight, BMI, quality of life (QoL), cost, and time saving. Furthermore, we included randomized controlled trials (RCTs), systematic reviews (SRs), meta-analyses (MAs), and clinical trials (CTs) for a comprehensive analysis. This systematic meta-review was based on PRISMA (Preferred Reporting Items for Systematic Reviews and Meta-Analyses) guidelines [12].

## Methods

### Search Strategy

Publications were systematically identified by searching the Cochrane Library, MEDLINE via PubMed, Web of Science Core Collection, EMBASE, and CINAHL databases for studies published between January 2008 and April 2020. First, we carried out a comprehensive literature search targeting T1DM, T2DM, gestational diabetes mellitus (GDM), and mixed studies involving different types of diabetes.

Search was conducted using the following keywords: (“diabetes mellitus”) AND (“telemedicine” OR “telemonitoring” OR “telemedicine”). Medical Subject Headings and Embase Subject Headings terms as well as title/abstract terms were used. This was supplemented with a manual search of the reference lists. Ultimately, studies that included both T1DM and T2DM patients were selected for inclusion in this systematic meta-review. The search strategies are shown in Multimedia Appendix 1. Studies were screened and selected by 2 independent reviewers, and any disagreement was resolved through consensus.

### Eligibility Criteria

Studies that met the following inclusion criteria were selected: peer-reviewed studies with full text; including T1DM and T2DM patients; addressing telemedical interventions for diabetes management; published in English or German; participants transfer data and receive appropriate feedback from health care professionals; and study designs: RCTs, SRs, MAs, and CTs. Qualitative and quantitative studies were considered. Telemedicine was defined as “remote acquisition, recording and transmission of patient data via a telecommunications system to a health care provider for analysis and decision making” [8]. Therefore, videoconferences, telephone calls, asynchronous communication by emails, SMS text messaging, internet/web-based platforms, and mixed forms (eg, videoconferences and emails) were included.

On the basis of this, the following studies were excluded: poster, comments, letters, study protocols, proceedings papers, studies that did not specify the types of diabetes of the population, studies providing pooled data with patients diagnosed with GDM, studies providing description of technologies only; studies conducted on smartphone/mobile apps (we analyzed these separately in another research paper because of the different nature of the technology), pooled data with other technologies, duplicates, and papers focusing on prevention or diagnosis.

### Data Extraction

Study characteristics (year of publication, study region, and design), patient characteristics (type of diabetes), intervention details (outcome measures, duration, intervention and control group, sample size, and information and communication technologies used), and main results and author’s conclusions were extracted.

### Outcomes of Interest

The primary outcome was HbA_1c_, and the secondary outcomes were FBG, BP, body weight, BMI, health-related quality of life (HRQoL), diabetes-related quality of life (DRQoL), cost effectiveness, and time saving.

### Data Synthesis and Analysis

A previously developed scheme to structure the constituent studies according to intervention types was used for data synthesis and analysis. On the basis of the information and communication technologies used between health care professionals and diabetes patients, we chose 4 types of intervention: *real-time video interventions*, *real-time audio interventions*, *asynchronous interventions*, and *combined interventions*.

*Real-time video* encompasses synchronous communication that takes place face to face, whereas *real-time audio* also covers synchronous communication, which occurs via telephone calls. *Asynchronous* depicts time-shifted communication, for example, using emails, server, SMS text messaging, and internet-/web-based platforms. The last category *combined interventions* combines *real-time* and *asynchronous* communication and accordingly includes elements from both forms.

### Assessment of Risk of Bias

The quality appraisal of the studies for the assessment of the risks of bias was carried out using 3 different validated instruments, as we included different study designs. For SRs and MAs, we applied *A MeaSurement Tool to Assess systematic Reviews* (AMSTAR 2). For RCTs and CTs, the *Effective Public Health Practice Project* (EPHPP) was applied. Moreover, the *National Institute for Health and Care Excellence* (NICE) quality appraisal checklist was deployed for qualitative studies. The tools grade the study quality into (critically) low/weak, moderate, and high/strong.

## Results

### Characteristics of the Included Studies

A systematic literature search identified 1647 citations. After duplicates were removed, we screened 1116 citations and excluded 875 ineligible papers based on their titles and abstracts. After assessing 241 studies with full text and excluding 72 inappropriate publications based on our inclusion and exclusion criteria, we manually searched reference lists and yielded 184 studies that covered T1DM, T2DM, and GDM. Finally, we included 31 suitable studies that comprised T1DM and T2DM patients in this systematic meta-review.

We identified 21 SRs and MAs, 8 RCTs, 1 non-RCT, and 1 qualitative study. SR and MA aside, 3 trials were conducted in the United States, 1 in Taiwan, 2 in the United Kingdom, 1 in Greece, 1 in Australia, and 1 in Israel.

In general, 8 SRs/MAs were rated with high, 12 with moderate and 1 with critically low quality based on AMSTAR 2. Furthermore, of 9 trials that were assessed using EPHPP, 6 were high/strong, 2 were moderate, and 1 was weak in quality. The qualitative study was assessed using the NICE checklist for qualitative studies, which resulted in high (++) quality.

Of the 10 identified intervention studies (without SRs and MAs), 3 were categorized as *real-time video interventions*, 1 as *real-time video and audio intervention*, 4 as *asynchronous interventions*, and 2 as *combined interventions* (real-time and asynchronous communication). Interestingly, none of the studies examined pure *real-time audio intervention*.

### Overview of Presentation of Findings

The characteristics of the publications are summarized in Table 1 and Table 2. The search and selection protocol is allocated as a PRISMA flowchart in Multimedia Appendix 2 [12]. A summary of the studies, their characteristics, intervention details, main findings, and conclusions are provided in Multimedia Appendix 3 [5,13-42]. All studies, except for the qualitative study, were in a controlled design. Multimedia Appendix 4 summarizes the quality assessments using different tools.

**Table 1:** Baseline characteristics of all studies (n=31).

Characteristics		Value, n (%)
**Study design**		
	SRs^a^ & MAs^b^	21 (67)
	RCT^c^	8 (25)
	Non-RCT	1 (3)
	Qualitative study	1 (3)
**Year**		
	2008-2011	3 (9)
	2012-2014	12 (38)
	2015-2017	9 (29)
	2018-2020	6 (19)

^a^SR: systematic review.

^b^MA: meta-analysis.

^c^RCT: randomized controlled trial.

**Table 2:** Baseline characteristics of the individual studies (n=10, without systematic reviews and meta-analysis).

Characteristics		Individual studies, n (%)
**Location**		
	United States	3 (30)
	Taiwan	1 (10)
	United Kingdom	2 (20)
	Greece	1 (10)
	Australia	1 (10)
	Israel	1 (10)
**Intervention**		
	Real-time video	3 (30)
	Real-time video+audio	1 (10)
	Asynchronous	4 (40)
	Combined (real-time+asynchronous)	2 (20)

### Effects on Primary and Secondary Clinical Outcomes

We synthesized the effects on the clinical outcomes in Multimedia Appendix 5. Table 3 provides a summary of the *significant* intervention effects on the clinical outcomes (intra- and intergroup effects are listed). In some cases, positive effects were not significant, and some studies indicated obvious improvements, but *P* values were not available. The denominators do not add up to the total “n” (left-hand panel) because not every study examined only one outcome.

**Table 3:** Significant effects on primary outcome HbA_1c_ and secondary outcomes (intra- and intergroup).

Study/outcome	HbA_1c_^a^	FBG^b^	BP^c^	Body weight	BMI	DRQoL^d^	HRQoL^e^	Cost	Time-saving	N/S^f^ or significance not available
SR^g^ & MA^h^ (n=21)	(13/17)^i^ +^j^	—^k^	(2/4) +	—	—	—	—	(2/5) +	N/A	—
Real-time video (n=3)	—	—	—	—	—	—	—	—	—	✓^l^
Real-time audio+video (n=1)	—	—	—	—	—	—	—	—	—	✓
Asynchronous (n=4)	(1/3) +	—	(1/1) +	—	(1/1) +	—	—	—	—	—
Combined (n=2)	—	—	—	(1/1) +	N/A	(1/1) +	—	—	—	—

^a^HbA_1c_: glycated hemoglobin A_1c_.

^b^FBG: fasting blood glucose.

^c^BP: blood pressure.

^d^DRQoL: diabetes-related quality of life.

^e^HRQoL: health-related quality of life.

^f^N/S: not significant.

^g^SR: systematic review.

^h^MA: meta-analysis.

^i^Number of publications with significant intervention effects versus number of all studies (inclusive significant effects that were not long term).

^j^+: outcome improvement.

^k^Missing data.

^l^N/S or significance not available.

#### SR and MA (n=21)

##### HbA_1c_ (n=17)

In general, all investigated SRs and MAs reported positive effects on HbA_1c_ values [5,13-28]. Most studies (n=13, 76.5%; high quality and moderate quality) showed significant improvements in HbA_1c_ concentrations. Significant reductions based on pooled T1DM and T2DM patients data ranged from −0.22% weighted mean difference (WMD; 95% CI −0.28 to −0.15; *P*<.001) by Wu et al [21] (moderate quality) to −0.64% mean difference (95% CI −1.01 to −0.26; *P*<.001) by So and Chung [17] (moderate quality). According to Faruque et al [13] (high quality), the intervention effect on HbA_1c_ was highest in studies with high baseline HbA_1c_ values and in interventions with asynchronous communication via web portals or text messaging. Toma et al [20] (high quality) also indicated the greatest significant reduction in asynchronous *internet only interventions* (−0.51%; 95% CI −0.68 to −0.34; *P*<.001). Su et al [18] (high quality) concluded that interventions with a duration of 6 months or less showed a greater decline in HbA_1c_ values (Hedge g=−0.56%; *P*<.001). Tao and Or [24] (high quality) also reported the greatest improvement in short-term interventions lasting 3 months or less (−0.54%, 95% CI −0.80 to −0.28; *P*<.001).

##### BP (n=4)

Overall, all studies (moderate quality and high quality) mentioned positive effects on BP [15,20,21,26]. Wu et al [21] (moderate quality) and Toma et al [20] (high quality) reported significant decreases in systolic and diastolic BP values compared with usual care: systolic BP WMD −1.92 mm Hg (95% CI −2.49 to −1.34; *P*<.001), diastolic BP WMD −1.31 mm Hg (95% CI −2.39 to −0.23; *P*<.001) [21], systolic BP −3.47 mm Hg (95% CI −5.0 to −1.94; *P*<.001), and diastolic BP −1.84 mm Hg (95% CI −2.98 to −0.70; *P*=.11) [20].

##### Body Weight (n=1)

Jong et al [26] (moderate quality) described that one telemedical intervention had positive effects on patients’ body weight, but no significance was reported.

##### BMI (n=3)

Studies by Wu et al [21] (moderate quality), Hu et al [14] (moderate quality), and Marcolino et al [15] (moderate quality) found positive effects of telemedical interventions on BMI, but were not statistically significant. Wu et al [21] (moderate quality) reported a difference between the telehealth and usual care groups in controlling BMI (WMD=−0.14 kg/m² (95% CI −1.13 to 0.68; *P*=.79). Hu et al [14] (moderate quality) outlined a reduction of 0.27 kg/m² (95% CI 0.86 to −0.31; I²=40%; *P*=.35).

##### DRQoL and HRQoL (n=3)

Two studies (66.7%) by Polisena et al [16] (high quality) and Wu et al [21] (moderate quality) concluded overall positive effects on DRQoL as well as HRQoL (not significant [21] and significance was not reported [16]), whereas Faruque et al [13] (high quality) found no effects on QoL.

##### Cost Effectiveness (n=5)

According to 4 papers (1 high quality, 2 moderate quality, and 1 critically low quality; 80.0%), telemedical interventions can be viewed cost effectively [5,23,29,30]. Tchero et al [5] (high quality) noted an incremental cost-effectiveness ratio (ICER) in 3 studies of US $490, US $29,869, and US $464, per capita for each unit reduction in HbA_1c_. Lee and Lee (moderate quality) [29] found a moderate cost-effectiveness in 7 telephone interventions of US $4744.32- US $86,276.50/quality-adjusted life year (ICER). Only Teljeur et al [31] (moderate quality) reported that telemedicine was not cost effective at all (based on 3 studies).

##### Barriers and Enablers (n=1)

MacDonald et al [32] (moderate quality) identified poorly designed interfaces as barriers and highly automated data entry and transmission, support by health care professionals and family, integration of users in the design process, and reliable technology as enablers for the implementation of communication technologies in diabetes management.

#### Real-Time Video Interventions (n=3)

##### HbA_1c_ (n=2)

The trials by Sood et al [33] (moderate quality) and Kearns et al [34] (weak quality) found no significant improvements in HbA_1c_ values (*P*>.05). Sood et al [33] (weekly video conferences) reported a decrease in HbA_1c_ (intervention −1.01% vs usual care −0.68%; *P*=.19).

##### BP (n=1)

Sood et al [33] (moderate quality) reported slightly, not significantly, increased BP values (+3.8 mm Hg) in the intervention group (*P*=.02).

##### Process Analysis of Video Consultations (n=1)

Fatehi et al [35] (high quality) analyzed video consultations qualitatively and found that health care professionals were confident with their feedback for diabetes patients via videoconferencing.

#### Real-time Audio+Video Intervention (n=1)

##### HRQoL (n=1)

According to a high quality study by Young et al [36] (combined telephone and videoconferencing intervention), physical and mental health clearly improved in the intervention group compared with usual care, but the difference was not statistically significant (*P*<.05).

#### Asynchronous Interventions (n=4)

##### HbA_1c_ (n=3)

In general, all RCTs (moderate quality and high quality) found reductions in HbA_1c_ values [37-39]. In addition, 2 RCTs reported significant improvements: Chen et al [37] (moderate quality) noted a significant improvement in the intervention group (*P*=.02), and Fountoulakis et al [38] (high quality) outlined a significant reduction in the intervention group at 3 (7.1±1.0%; *P*<.001) and 6 months (6.9±0.9%; *P*<.001), compared with the control group.

##### BP (n=1)

In a high quality RCT by Earle et al [40], systolic BP fell significantly in the intervention group (−6.5 mm Hg; 95% CI −0.8 to −12.2; *P*=.03) and remained unchanged in the control group (*P*=.57).

##### BMI (n=1)

According to Fountoulakis et al [38] (high quality), a significant BMI reduction (*P*<.05) was observed in the intervention participants at 6 months as well as at 6-months off telemonitoring compared with baseline, whereas intergroup analysis was not significant.

#### Combined Interventions (n=2)

##### HbA_1c_ (n=2)

In 2 high quality RCTs by Leichter et al [42] and Boaz et al [41], the authors observed no significant differences in post treatment HbA_1c_, and the values slightly increased in the intervention groups.

##### FBG (n=1)

Boaz et al [41] (high quality) observed a nonsignificant decrease in FBG values compared with the control group.

##### BP (n=1)

Leichter et al [42] (high quality) reported a nonsignificant increase in systolic BP in the intervention group.

##### Body Weight (n=2)

Although Leichter et al [42] (high quality) showed a significantly greater reduction in body weight in the intervention group (−5.2 vs −0.7 pounds; *P*=.04), Boaz et al [41] (high quality) found a slight, not significant, weight gain in treatment subjects.

##### BMI (n=1)

According to Leichter et al [42] (high quality), BMI decreased in the intervention group, with no significant between-group differences at 12 months [42].

##### DRQoL (n=1)

Boaz et al [41] (high quality) reported that intervention patients reported significantly greater posttreatment experiences of improved QoL [41]. The DRQoL measures *being clinically symptom-free* (71% vs 11%; *P*=.003), *having no hypoglycemic events* (82% vs 17%; *P*<.001), and *having no hyperglycemic events* (65% vs 17%; *P*=.004) were significantly more frequent in the intervention group.

##### Time Saving (n=1)

According to Leichter et al [42] (high quality), the clinician time requirements for intervention subjects were reduced by 40% (significance not reported).

### Differences Between T1DM and T2DM

In total, 5 studies (3 high quality and 2 moderate quality) specifically examined differences in treatment effects between T1DM and T2DM patients [5,18,20,22,27]. Of these, 4 studies [5,18,20,27] reported smaller effects for T1DM, and 1 publication by Hanlon et al [22] (moderate quality) observed significantly improved glycemic control (HbA_1c_) in T2DM but not T1DM.

Su et al [18] (high quality) described that telemedicine was most effective in T2DM patients (Hedge g=−0.63; *P*<.001), whereas the effect was smaller for T1DM patients (Hedge g=−0.27; *P*=.03) and for T1DM and T2DM combined (Hedge g=−0.34; *P*=.003). The difference was statistically significant between T1DM and T2DM (Q statistics=4.25; *P*=.04). Tchero et al [5] (high quality) observed similar findings. Patients diagnosed with T2DM experienced a significantly greater reduction in HbA_1c_ concentrations compared with those diagnosed with T1DM (Hedge g=−0.48, *P*=.001 vs −0.26, *P*=.05; Q=1935.75, *P*<.001). In accordance with this, Toma et al [20] (high quality) reported a significantly smaller effect in T1DM (−0.12%; 95% CI −0.32 to −0.08; *P*=.26) than in T2DM (−0.55%; 95% CI −0.68 to −0.42; *P*<.001). Kitsiou et al [27] (moderate quality) outlined similar findings, but this finding was not significant. Telemedical interventions improved glycemic control (HbA_1c_) compared with usual care: mean difference −0.8% (95% CI −1.11. to 0.5; n=280 patients; *P*<.05) for patients with T2DM and mean difference −0.3% (95% CI 0.0 to 0.5; n=645 patients; *P*>.05) for patients with T1DM.

## Discussion

### Principal Findings

In total, telemedical interventions might improve diabetes management in studies that pooled T1DM and T2DM participants overall and probably improve HbA_1c_ values, which corresponds to our first hypothesis. T2DM patients could benefit more from telemedical interventions than T1DM patients regarding lowering HbA_1c_ levels, but limitations must be taken into account, such as higher baseline HbA_1c_ values, short duration, small effect sizes, and partially small cohorts.

T1DM and T2DM have contrasting pathogenesis, which also results in appropriate therapy recommendations with different priorities [1]. Patients with T1DM are insulin deficient and require insulin therapy, and patients with T2DM are insulin resistant [1]. Although lifestyle changes are part of both T1DM and T2DM management, the focus of T1DM is on insulin therapy, whereas T2DM is, besides medication, more focused on nutrition regarding overweight and regular exercise [10]. Telemedical diabetes management may be more effective in T2DM patients by addressing modifiable factors such as nutrition and exercise [10]. Furthermore, T1DM patients are a unique group with special needs [11]. They are rather younger people, although both types of diabetes can occur at any age [1]. Management of T1DM in childhood and adolescence is both a medical and psychosocial challenge, as adolescence is a very vulnerable period [11]. For example, hormonal fluctuations and complications related to excessive insulin daily doses (eg, menstrual irregularities and weight gain) [43] as well as resistance to parents and health care services [11] play a role. These special features might have an impact on the differences between the types of diabetes. The findings further illustrate the importance of tailoring diabetes management to the patient and his/her needs [32].

### Primary Outcome HbA_1c_

HbA_1c_ was certainly the most investigated outcome. Numerous studies with high quality and moderate quality showed that telemedical interventions effectively reduced HbA_1c_ values significantly, up to −0.64% mean difference (95% CI −1.01 to −0.26; *P*<.001) by So and Chung [17]. Few *real-time video interventions* with moderate quality and WEAK QUALITY indicated clear but not significant improvements in HbA_1c_ levels. The *asynchronous interventions* showed significant HbA_1c_ reductions in some moderate quality and high quality studies. In contrast, few *combined interventions* generally miscarried to reduce HbA_1c_ values effectively, but we only analyzed 2 appropriate trials.

Our findings regarding more effective HbA_1c_ improvements compared with usual care are an important success for digital diabetes therapy because strict glycemic control is critical for both T1DM and T2DM patients [1,44]. Optimal glycemic control reduces and prevents micro- and macrovascular diabetic complications [44]. Microvascular complications such as nephropathy, retinopathy, and neuropathy as well as macrovascular events, such as cardiovascular disease and stroke, are closely associated with T1DM and T2DM, respectively [1,45]. The *legacy effect* showed that an HbA_1c_ value in the target area is predominant at an early stage because increased HbA_1c_ concentrations in the first year after a T2DM diagnosis increase the risk of micro- and macrovascular events in the long term. Even patients with HbA_1c_ levels between 7% and <8% had an increased risk compared with patients with HbA_1c_ levels below 6.5%.

*BP*: Telemedical interventions improved BP values in several moderate quality and high quality studies. There were positive significant effects on BP through an *asynchronous* high quality intervention. Clinical studies have shown an association between T2DM, vascular disease, and the present risk factor hypertension [45]. Therefore, the observed positive tendencies should be investigated further.

*Body weight*: Telemedical interventions demonstrated positive tendencies in terms of body weight improvement, which is particularly evident in a moderate quality review, but the number of identified studies was rare. These positive tendencies should be examined more closely.

*FBG*: One *combined intervention* (synchronous and asynchronous communication) examined the outcome FBG and indicated a nonsignificant decrease in FBG values.

QoL: Several studies with high quality and moderate quality indicated positive effects of telemedicine on DRQoL and HRQoL. The high quality real-time audio and video intervention significantly improved physical and mental health. Likewise, the high quality combined intervention significantly enhanced DRQoL.

*Cost*: In general, 80% (4/5) of the SRs and MAs demonstrated cost effectiveness of telemedical interventions. Telemedicine appears to be a cost-effective option in the context of diabetes management with limitations, as cost effectiveness is related to country conditions and settings.

*Time saving*: Although only 1 study examined this outcome, this high quality study showed that the clinician time could be reduced.

Overall, important outcomes with little evidence available, such as FBG, body weight, cost, and time saving in particular, all showed positive tendencies and should be investigated further.

*Type of intervention*: To determine the types of telemedical interventions that are particularly effective and successful, we previously developed a scheme that divides the interventions into 4 categories. Overall, only a few studies could be identified for these categories. Interestingly, we could not identify any pure *real-time audio interventions* (communication via telephone calls only), just 1 study that included both real-time video and audio communication. *Real-time video interventions * showed obvious improvements in HbA_1c_ values.*Asynchronous interventions* significantly improved HbA_1c_ levels, BP, and BMI. In addition, *combined interventions* showed potential in improving BMI and DRQoL. No type of intervention was clearly superior. Some SRs and MAs indicated that asynchronous interventions (web portal, email, and internet) were more successful. The type of intervention or the form of communication should be adapted to the needs of diabetes patients [32]. A poorly designed interface can be a barrier for the patient and impair the success of the digital intervention [32]. Reliable technologies and support by health care professionals and families promote the success of telemedical approaches [32]. When patients believe that the technology is useful and offers feedback and flexibility, they are more likely to adopt this new option [32]. In total, we assume that telemedical approaches improve compliance, empower patients, and enable more intensive therapy as well as effective disease management on the part of health care professionals and patients.

From a clinical point of view, it is important to know the effect size that only results from modifying the communication level by using telemedicine. In clinical practice, these effects must be known and added to the therapeutic effects (eg, by insulin). This is also important to provide evidence-based recommendations. In addition, the relatively short duration of the studies must be taken into account. In principle, further studies with longer duration and larger cohorts are necessary to analyze the effect sizes in more detail.

The strengths of this systematic meta-review lie in the scheme used to categorize the telemedical interventions, the consideration of numerous study designs, the analysis of several clinically important outcomes, and the investigation of specific differences in the intervention effects between T1DM and T2DM patients.

### Limitations

Some limitations may have affected our findings. There were large variations in the types of telemedical technology used in the SRs and MAs, as telemedicine is a broad term with different definitions. The number of constituent studies in our 4 categories for the technologies used and their sample sizes were small for a number of outcomes measured. The trials comprised diverse interventional approaches, durations, and frequencies of contact with health care clinicians. Furthermore, many outcome measurements led to insignificant results, which may indicate methodological weaknesses. Some of the SRs and MAs that examined the differences between T1DM and T2DM indicated possible confounding factors that must be taken into account. A high baseline HbA_1c_ can lead to a larger reduction in HbA_1c_ during any kind of intervention.

### Comparison With Prior Work

The findings of this systematic meta-review are overall consistent with other SRs and MAs on telemedicine for diabetes management. An effective and significant reduction in HbA_1c_ values in patients diagnosed with T1DM or T2DM through telemedicine was also reported by numerous other authors in evidence synthesis [5,13,46]. Wu et al [21] also demonstrated the positive impact of telemedical diabetes management on BP, BMI, and QoL in their MA on telehealth for managing diabetes. Tchero et al [5] (clinical effectiveness of telemedicine based on 42 RCTs) and Su et al [18] (impact of telemedicine on HbA_1c_, including 55 RCTs) reported obviously smaller effects for T1DM patients on HbA_1c_ values than for T2DM patients.

### Conclusions

Taken together, this systematic meta-review demonstrated that telemedical interventions might be clinically effective in the management of populations consisting of T1DM and T2DM patients. The intervention effect on lowering HbA_1c_ values may be smaller for T1DM patients than for T2DM patients. Although evidence on the impact on BP, body weight, FBG, cost effectiveness, and time saving is smaller compared with HbA_1c_, potential is indicated in some publications. Although none of the intervention type we categorized—*real-time video/audio communication*, *asynchronous communication*, and *combined communication* (*real-time and asynchronous*) was superior, each type generally showed improvements in clinical diabetes management.

Further research is needed regarding the differences between T1DM and T2DM with regard to the improvement of BP, body weight, and FBG. Overall, improvement in FBG values should be investigated in more detail, as we only identified one study measuring this outcome. Our findings indicate that telemedical applications are promising in the context of diabetes therapy, but further studies with longer duration and larger cohorts are necessary.

## Appendix

Multimedia Appendix 1 Search strategies.

Multimedia Appendix 2 Search and selection protocol as PRISMA (Preferred Reporting Items for Systematic Reviews and Meta-Analyses) flowchart.

Multimedia Appendix 3 Overview of studies included in systematic meta-review.

Multimedia Appendix 4 Quality assessments.

Multimedia Appendix 5 Effects on primary outcome HbA_1c_ and secondary outcomes.

## Abbreviations

AMSTAR 2: A Measurement Tool to Assess Systematic Reviews
BP: blood pressure
CT: clinical trial
DFG: Deutsche Forschungsgemeinschaft
DM: diabetes mellitus
DRQoL: diabetes-related quality of life
EPHPP: Effective Public Health Practice Project
FBG: fasting blood glucose
GDM: gestational diabetes mellitus
HbA_1c_: glycated hemoglobin A1c
HRQoL: health-related quality of life
ICER: incremental cost-effectiveness ratio
MA: meta-analysis
MAST: model for assessment of telemedicine
NICE: National Institute for Health and Care Excellence
PRISMA: Preferred Reporting Items for Systematic Reviews and Meta-Analyses
QoL: quality of life
RCT: randomized controlled trial
SR: systematic review
T1DM: type 1 diabetes mellitus
T2DM: type 2 diabetes mellitus
WMD: weighted mean difference
