# Supplementary material for: Clinical Improvements by Telemedicine Interventions Managing Type 1 and Type 2 Diabetes: Systematic Meta-review
Source: J Med Internet Res. 2021 Feb 19;23(2):e23244. doi: 10.2196/23244 (PMC7935656; doi:10.2196/23244)
Supplement: Multimedia Appendix 2 [file jmir_v23i2e23244_app2.pdf]

Search and selection protocol as PRISMA flow chart (adapted from Moher et al. 2009).

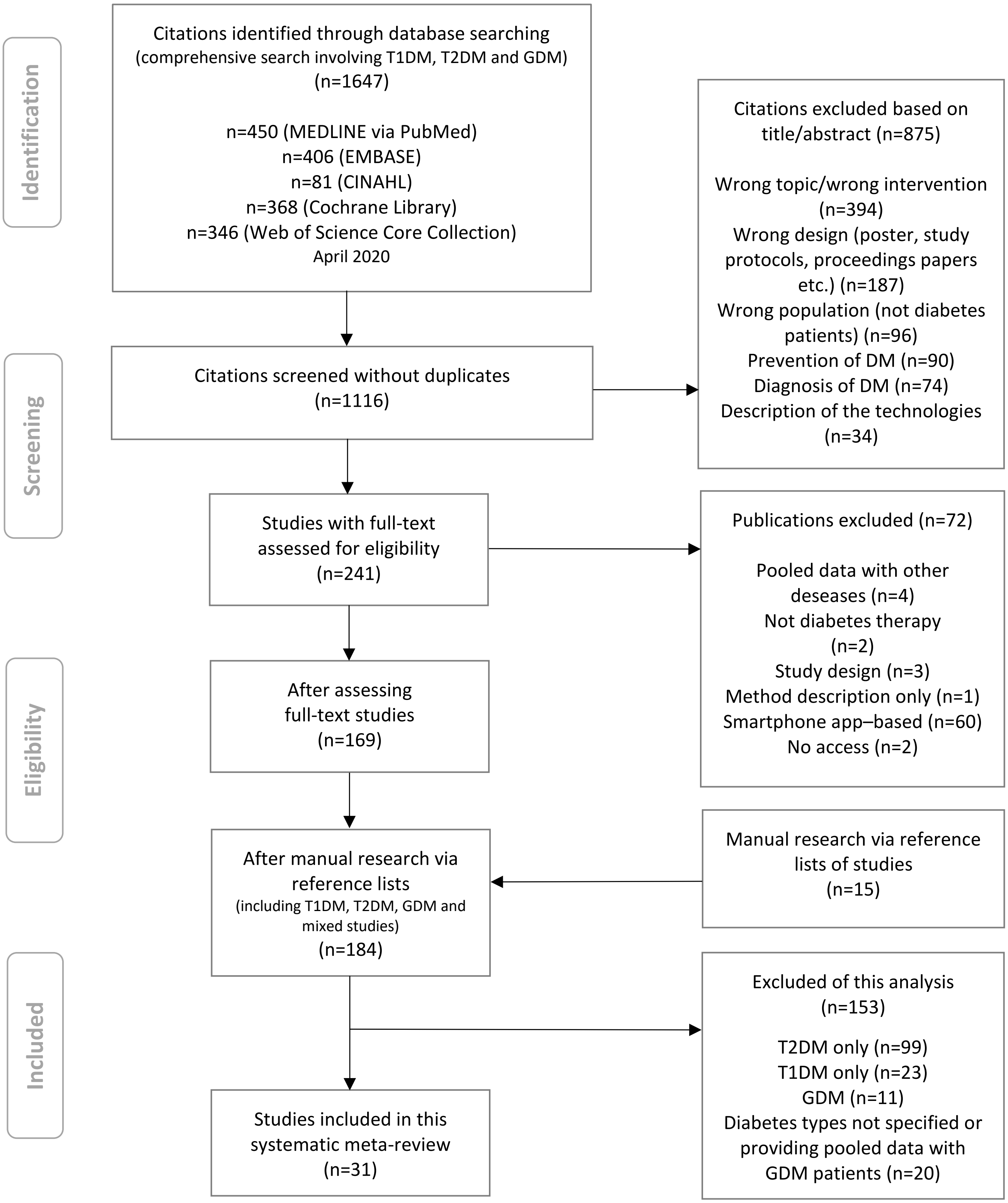

T1DM: type 1 diabetes mellitus, T2DM: type 2 diabetes mellitus, GDM: Gestational diabetes mellitus.
