# Supplementary material for: Clinical Improvements by Telemedicine Interventions Managing Type 1 and Type 2 Diabetes: Systematic Meta-review
Source: J Med Internet Res. 2021 Feb 19;23(2):e23244. doi: 10.2196/23244 (PMC7935656; doi:10.2196/23244)
Supplement: Multimedia Appendix 3 [file jmir_v23i2e23244_app3.pdf]

Overview of studies included in systematic meta-review.

Author, Year, Location	Topic	Study design	Participants/ included studies	Intervention and Control group	Main results	Overall value of intervention(s)
**(Faruque et al. 2017)**<br>**-**	Effect of telemedicine on glycated hemoglobin in diabetes	SR & MA of RCTs	RCTs (n=111); Patients (n=23,648)	Web portal (24%), customized "smart" device (14%), telephone for communication to provider (13%), smartphone application (8%), SMS (5%), email (3%), personal digital assistant (2%), automated voice reminder system (1%), computer software (1%), fax (1%), listserv (electronic mailing list to send group emails; 1%), customized patient-specific Web page (1%) or a call-me button (1%)	• Telemedicine achieved significant but modest reductions in HbA_{1c} in all 3 follow-up periods (difference in mean at ≤ 3 mo: −0.57%, 95% CI −0.74% to −0.40% [39 trials]; at 4–12 mo: −0.28%, 95% CI −0.37% to −0.20% [87 trials]; and at > 12 mo: −0.26%, 95% CI −0.46% to −0.06% [5 trials])<br>• In meta-regression analyses, the effect of telemedicine on HbA_{1c} appeared greatest in trials with higher HbA_{1c} concentrations at baseline, in trials where providers used Web portals or text messaging to communicate with patients and in trials where telemedicine facilitated medication adjustment<br>• Telemedicine had no convincing effect on QoL (DR and HR), mortality or hypoglycemia<br>• Significant improvement in Problem Areas in Diabetes Score (MD at 4-12 months: 2.86% CI 1.74 to 3.97, n=363 patients), three scores showed significant worsening (HRQoL)	Positive:<br>Compared with usual care, the addition of telemedicine, especially systems that allowed medication adjustments with or without text messaging or a Web portal, improved HbA_{1c} but not other clinically relevant outcomes
**(Hu et al. 2019)**<br>**Spain, Korea, China, US, Italy, Greece, India, Israel, Saudi Arabia, *P*oland, Canada**	Effect of telemedicine intervention on hypoglycaemia in diabetes patients	SR & MA of RCTs	RCTs (n=14); Patients (n=1,324)	Computer software (14%), internet–based monitor system (43%), and smart device (43%)	• Compared to usual care, telemedicine was found to reduce the odds of hypoglycaemia (odds ratio (OR)=0.42; 95% CI=0.29–0.59; I^2=32%; *P*<.00001)<br>• Clinical relevance declined in HbA_{1c} level compared to CG (mean difference=-0.28; 95% CI=-0.45 to -0.12; I^2=53%; *P*=.0005), but that telemedicine had no significant effect on BMI (mean difference=-0.27; 95% CI=0.86–0.31; I^2=40%; *P*=.35)	Positive:<br>The use of telemedicine was found to improve HbA_{1c} and reduce the risk of moderate hypoglycaemia but without significant difference in BMI
**(Lee and Lee 2018)**<br>**US, Canada, Australia, Singapore, UK, Norway, Finland, Greece, India**	Telemedicine Cost–Effectiveness for Diabetes Management	SR	n=14	Economic evaluations of telemedicine in diabetes care (Telemonitorig + Teleophthalmology screening + Telephone interventions)	• 2 studies examined the cost–effectiveness of telemonitoring<br>• Study 1: cost-effective method for improving patient QoL, with an estimated cost of €5,460.11/QALY for the Greek national health system, €1,776.70/QALY for the German, an €1,013.48/QALY for the Italian<br>• Study 2: cost of CAD$ 33,789/QALY using continuous glucose monitoring<br>• Overall, 7 studies exploring the use of telephone intervention in diabetes management reported moderate cost–effectiveness (inflation-adjusted ICER: $4,744.32–$86,276.50/QALY)	Positive<br>Use of telemonitoring and telephone reminders was cost-effective

Author, Year, Location	Topic	Study design	Participants/ included studies	Intervention and Control group	Main results	Overall value of intervention(s)
**(Macdonald et al. 2017)**<br><br>**North America (49%), Europe (38%) and Asia (11%)**	Enablers and barriers to using two-way information technology diabetes management	Descriptive SR	n=48	Trials for adults with diabetes where the patient used biomedical and/or communication technology to support their diabetes management	• Barriers included poorly designed interfaces requiring manual data entry and systems that lacked functionalities valued by patients<br>• Enablers included access to reliable technology, highly automated data entry and transmission, graphical display of data with immediate feedback, and supportive health care professionals and family members<br>• Users should be consulted in the design process and consideration given to theories of technology adoption to inform design and implementation<br>• Technology should be as automated, streamlined, mobile, low cost and integrated as possible	-
**(Marcolino et al. 2013)**<br><br>**US, Europe**	Telemedicine application in the care of diabetes patients	SR & MA	RCTs (n=13)	Telemedicine strategies included different combinations of transmission of monitoring data, videoconferencing, educational web site, educational reminders, email exchange, evidence–based message to the general physician, teleconsultation, telephone calls, short message service and forum discussion	• Telemedicine was associated with a statistically significant and clinically relevant absolute decline in HbA_{1c} level compared to control (mean difference -0.44% [-4.8 mmol/mol] and 95% CI -0.61 to -0.26% [-6.7 to -2.8 mmol/mol]; *P*<.001)<br>• LDL-c was reduced in 6.6 mg/dL (95% CI -8.3 to -4.9; *P*<.001), but the clinical relevance of this effect can be questioned<br>• No effects of telemedicine strategies were seen on systolic (-1.6 mmHg and 95% CI -7.2 to 4.1) and diastolic BP (-1.1 mmHg and 95% CI -3.0 to 0.8), *P*>.05<br>• Not significant reduction in BMI (2 studies)	Positive:<br>Telemedicine strategies combined to the usual care were associated with improved glycemic control in diabetic patients<br>No clinical relevant impact was observed on LDL-c and BP<br>Positive tendency BMI
**(Polisena et al. 2009)**<br><br>**US, Canada, Germany, *P*oland, Finland, Spain, South Korea, Italy, China**	Home telehealth for diabetes management	SR & MA	RCTs (n=26); Patients (n=5,069)	Home telemonitoring (21 studies) and telephone support (5 studies)	• Telemonitoring had a positive effect on glycaemic control [as measured by lower HbA_{1c} level] compared with usual care (weighted mean difference =-0.21; 95% CI -0.35 to -0.08) (S N/R)<br>• Home telehealth was favourable to usual care across studies for QoL (HR and DR) and patient satisfaction outcomes<br>• home telehealth helps to reduce the number of patients hospitalized, hospitalizations and bed days of care	Positive
**(So and Chung 2018)**<br><br>**Iran, South Korea, US**	Telehealth for diabetes self-management in primary health care	SR & MA	RCTs (n=7)	Telehealth on diabetes control self-management in primary health care settings	• All reported decreasing level of HbA_{1c}; overall effect was significant (Z=3.31,*P*=.0009); mean difference HbA_{1c} -0.64 [CI 95% -1.01, -0.26] with intervention n=415 and control n=438<br>• Fasting plasma glucose MD -0.26 [CI 95% -1.05, -0.53], *P*=.52 with intervention n=225 and control n=230	Positive

Author, Year, Location	Topic	Study design	Participants/ included studies	Intervention and Control group	Main results	Overall value of intervention(s)
**(Su et al. 2016)**<br><br>**US, Europa, Asia, Australia**	Does telemedicine improve treatment outcomes for diabetes?	MA	RCTs (n=55); Patients (n=9,258)	37 utilized a high-level intervention (device–based telemonitoring), while 18 utilized a low-level intervention (teleconsultation only)	• HbA_{1c}: Telemetry was most effective in T2DM patients (Hedges's g = -0.63, *P*<.001), whereas the effect was smaller for T1DM patients (Hedges's g = -0.27, *P*=.027) T1DM and T2DM combined (Hedges's g = -0.34, *P*=.0.003)<br>• Difference statistically significant between T1DM and T2DM (Q = 4.25, *P*=.04)<br>• Programs lasting 6 months or less showed greater reduction in HbA_{1c} levels (Hedges's g = -0.56, *P*<.001)<br>• Telemetry was more effective among patients of ages 40 or older (Hedges's g = -0.53, *P*<.001) than among patients younger (Hedges's g = -0.32, *P*=.024)	Positive:<br>Compared to conventional care, telemedicine is more effective in improving treatment outcomes, especially for those with T2DM
**(Su et al. 2015)**<br><br>**US, Europe, Asia, Australia, Canada**	Does nutritional counseling in telemedicine improve treatment outcomes for diabetes?	SR & MA	n=92	Nutritional counseling as part of a telemedicine program (text messages, e-mail, videoconference, telephone)	• Telemedicine programs that include a nutritional component show similar effect in diabetes management as those programs that do not<br>• Subgroup analysis reveals that nutritional intervention via SMS such as email and text messages is at least as equally effective in reducing HbA_{1c} when compared to videoconference or telephone<br>• Overall, the telemedicine interventions in the 92 studies significantly reduced HbA_{1c} (Hedges's g=0.676, SE=0.060, *P*<.001)	Positive:<br>Significant decline in HbA_{1c} values, Inclusion of nutritional counseling as part of a telemedicine program does not make a significant difference to diabetes outcomes
**(Tchero et al. 2019)**<br><br>**US, Europe, Asia, Australia**	Clinical effectiveness of telemedicine in diabetes mellitus	MA	RCTs (n=42); Patients (n=6,170)	8 teleconsultation<br>34 telemonitoring	• Mean reduction in HbA_{1c} was significantly higher in telemedicine groups (Hedges' g=-0.37, *P*=.001)<br>• T2DM patients experienced a higher reduction in HbA_{1c} compared to T1DM (Hedges' g=-0.48, *P*=.001 vs. -0.26, *P*=.05; Q= 1935.75, *P*=.0001)<br>• Programs lasting >6 months significantly greater reduction in HbA_{1c} levels (Hedges' g=-2.24 vs. -0.66, *P*=.001)<br>• Internet–based teleconsultation (or through phone) is less cost-effective than remote monitoring of one's blood glucose levels<br>• 3 studies reported costs:<br>○ ICER, incremental cost-effectiveness ratio: \$490, \$29,869, and \$464 per capita for each unit reduction in HbA_{1c}	Positive:<br>Telemedicine interventions are more effective than usual care in managing diabetes, especially T2DM

Author, Year, Location	Topic	Study design	Participants/ included studies	Intervention and Control group	Main results	Overall value of intervention(s)
**(Toma et al. 2014)**<br><br>**Europe, US, Korea, Canada, Taiwan, India etc.**	Online social networking services in the management of patients with diabetes mellitus	SR & MA	RCTs (n=34)	Social networking services (SNS): real-time communication and feedback between patients and health care professionals	• Significant reduction in HbA_{1c} favouring the intervention group, WMD 0.46% (95% CI [-0.58, -0.34], *P*<.00001), heterogeneity was high (I^2 = 74%)<br>• Interventions significantly improved systolic and diastolic BP<br>○ Systolic: -3.47mmHg [95% CI -5.01, -1.94], *P*<.00001<br>○ Diastolic: -1.84mmHg [95% CI -2.98, -0,70], *P*=0.112<br>• T2DM patients had a significantly greater reduction in HbA_{1c} than T1DM<br>○ T1DM: -0.12% [95% CI -0.32, -0.08], *P*=.26<br>○ T2DM: -0.55% [95% CI -0.68, -0.42], *P*<.00001<br>• Highest reduction HbA_{1c} in Internet only interventions -0.51% [95% CI -0.68, -0.34], *P*<.00001<br>• Highest reduction HbA_{1c} in duration ≤3 months -0.54 [95% CI -0.80, -0.28), *P*<.00001	Positive:<br>Feasible approach to improving glycaemic control, particularly in T2DM patients
**(Wu et al. 2018)**<br><br>**US, Europe**	Evaluation of the clinical outcomes of telehealth for managing diabetes	MA	RCTs (n=19); Patients (n=6,294)	Telehealth intervention in most of the selected trials involved self-monitoring of blood glucose and data transmission, either manually or electronically, with feedback	• HbA_{1c} levels in the telehealth group were<br>• significantly lower than those in the usual care group (weighted mean difference = -0.22%; 95% CI, -0.28 to -0.15; *P*<.001)<br>• Statistically significant decrease in systolic BP (weighted mean difference=-1.92; 95% CI, -2.49 to -1.34; *P*<.001) and diastolic BP (weighted mean difference =-1.31; 95% CI, -2.39 to -0.23; *P*<.001) in the telehealth group compared to the usual care group<br>• No significant difference between the telehealth and the usual care group in controlling BMI (weighted mean difference =-0.14; 95% CI, -1.13 to 0.68; *P*=.79)<br>• QoL improved but not significant (2 studies)	Positive:<br>Targeting patients with higher HbA_{1c} (≥9%) levels and delivering more frequent intervention (at least 6 times 1 year) may achieve greater improvement
**(Hanlon et al. 2017)**<br><br>**-**	Telehealth interventions to support self-management: diabetes, heart failure, asthma, chronic obstructive pulmonary disease, and cancer	Systematic Meta-review	Type 1 (n=6)<br>Type 2 (n=2)<br>Mixed (n=19)	Telehealth interventions to support self-management on disease control and health care utilization (Telemonitoring)	• The highest-weighted reviews showed that blood glucose telemonitoring with feedback and some educational and lifestyle interventions effectively and significantly improved glycemic control (HbA_{1c}) in type 2, but not type 1 diabetes<br>• Evidence for telephone support was more limited, although it may be effective as part of intensive intervention	Positive for diabetes:<br>Telehealth is a safe option, particularly in heart failure and T2DM

Author, Year, Location	Topic	Study design	Participants/ included studies	Intervention and Control group	Main results	Overall value of intervention(s)
**(Siriwardena et al. 2012)**<br><br>**-**	Telemedicine interventions in diabetes care	SR	n=27	Telemedicine tools: videoconferencing (30%), Mobile phones (37%), telephone calls (33%), Feedback letters –based on telementoring (7%)	• HbA_{1c} improvement in 23 studies, which was significant in 12 out of 23 (44%)<br>• All of the studies (n=2) that analysed cost-effectiveness reported beneficial effects	Positive:<br>Telemedicine appears to be a promising alternative to conventional therapy
**(Tao and Or 2013)**<br><br>**US, Canada, Europe**	Effects of self-management health information technology (SMHIT) on glycaemic control for patients with diabetes	SR & MA of RCTs	RCTs (n=43)	3 categories: computer–based SMHIT, mobile phone–based SMHIT and other SMHIT types	• Significant reduction in HbA_{1c} compared to usual care, pooled standardized mean difference of -0.30% (95% CI -0.39 to -0.21, *P*=.001)<br>• Effect is significantly greater when the technology is a web–based application, when a mechanism for patients' health data entry is provided and the technology is operated in the home or without location restrictions	Positive:<br>Review supports the use of SMHITs as a self-management approach to improve glycaemic control
**(Walker et al. 2017)**<br><br>**-**	Home telemedicine interventions for the treatment of older adults with diabetes	SR	n=6	Home telemedicine interventions	• Case management, education, closed-loop feedback and communication, home telemonitoring devices or units, and motivational interviewing or coaching can effectively decrease admissions, costs per person per year, mortality, and cognitive decline in older adults with diabetes	Mildly positive:<br>Low quality studies
**(Baron et al. 2012)**<br><br>**Asia, Europe, US**	Impact of mobile monitoring technologies on glycosylated hemoglobin in diabetes	SR	n=24	Mobile phone transmission; feedback automated, via text message, web-portal or letter.	• 10 of 13 studies in T2DM and 4 of 7 studies on T1DM found interventions to lead to benefits (HbA_{1c})<br>• Studies without health care professional feedback led to improved HbA_{1c}, suggesting feedback might not be necessary for intervention success	Positive
**(Jong et al. 2014)**<br><br>**US, Korea**	Effects on health behavior and health outcomes of internet–based asynchronous communication between health providers and patients with a chronic condition	SR	n=4 (T1DM/ T2DM)	internet-Based Asynchronous Communication (different components, such as peer-support groups, sharing medical records, self-management programs, and patient portals)	• Improvements were shown in HbA_{1c} level (n=4 studies), and in body weight, cholesterol, HDL and blood pressure (each n=1 study), no further details reported	Positive
**(Kitsiou et al. 2017)**	Effectiveness of mHealth interventions	SR	SRs (n=15)	Mobile technologies such as cellular phones, personal digital assistants, tablet PCs (e.g. iPads) for monitoring; feedback via text messaging, web browsing, e-mail, and videos	• Interventions improved glycemic control (HbA_{1c}) compared to standard care or other non-mHealth approaches by as much as MD -0.8% (95% CI: -1.11, -0.5%), n=280 patients, *P*<.5 for patients with T2DM and 0.3% (95% CI: 0.0, -0.5%), n=645 patients, *P*>.5 for patients with T1DM, at least in the short-term (≤12 months)	Positive:<br>Especially T2DM

Author, Year, Location	Topic	Study design	Participants/ included studies	Intervention and Control group	Main results	Overall value of intervention(s)
**(Teljeur et al. 2017)**<br><br>**-**	Economic evaluation of chronic disease self-management for people with diabetes	SR	Telemedicine (n=11)	Self-management telemedicine interventions; technologies not specified	• Telemedicine-type interventions were more expensive than usual care and potentially not cost-effective<br>• Telemedicine for people with diabetes was not cost-effective below willingness-to-pay thresholds equivalent to $65,000 per QALY (3 studies)	Not cost-effective
**(Suksomboon et al. 2014)**<br><br>**Thailand**	Impact of phone call intervention on glycemic control in diabetes patients	SR & MA of RCTs	RCTs (n=5); Patients (n=953)	Telephone interventions	• Not significant improvement: pooled mean difference in HbA_{1c} -0.38% (95%CI -0.91, 0.16%), *P*=.17	May still have potential benefits especially for low-and middle-income countries
**"Real-time video interventions" (n=3)**						
**(Sood et al. 2018)**<br><br>**US**	Telemedicine consultation for patients with diabetes mellitus	RCT	IG (n=199)<br>CG (n=83)	IG: weekly telemedicine consultation, via videoconference<br>CG: weekly clinic visit<br><br>18 months	• Both groups showed decrease in HbA_{1c}, with no statistical difference between groups (telemedicine consultation -1.01% vs usual consultation -0.68%, *P*=.19)<br>• Better satisfaction with telemedicine consultations and positive reviews from the primary health care teams<br>• Systolic BP +3.8 mmHg in IG and -3.7 mmHg in CG (*P*=.016)<br>• LDL -6.3 mg/dL in IG and -14.3 mg/dL in CG (*P*=.30)<br>• Serum creatinine +0.06 mg/dL in IG and +0.12 mg/dL in CG (*P*=.27)	Similar clinical outcomes
**(Kearns et al. 2012)**<br><br>**US**	Group diabetes education administered through telemedicine: tools used and lessons learned	Non-rando-mized controlled clinical trial	IG (n=27)<br>CG (n=39)	IG: Diabetes education through real-time teleconferencing 2 3-h sessions, sessions were followed in 3 months by a 3-h follow-up class<br>CG: face-to-face group<br><br>3 months	• HbA_{1c} test improved in both groups, but difference was not significant between groups or within groups<br>• Each group had significant improvements in scores on the Problem Areas In Diabetes survey, which is a measure of emotional functioning in diabetes<br>• Diabetes treatment satisfaction improved in CG but not in IG<br>• Although the CG had significantly higher scores in the Diabetes Treatment Satisfaction Questionnaire, the IG was highly satisfied with the services provided	

Author, Year, Location	Topic	Study design	Participants/ included studies	Intervention and Control group	Main results	Overall value of intervention(s)
**(Fatehi et al. 2013)**<br><br>**Australia**	Telemedicine for clinical management of diabetes – a process analysis of video consultations	Qualitative	Video consultations (n=56)	Analysis of 56 video consultations by two endocrinologists via questionnaire<br><br>5 months	• Out of 56 consultations, the specialists indicated the need to perform a physical examination for 12 patients that was not possible remotely<br>• They requested an in-person (face-to-face) visit for three patients<br>• They believed that in 34% of the cases they could have made a better decision if the consultation had been in-person	Positive:<br>Video consultation can substitute for a large proportion of in-person specialist consultations for people with diabetes who are referred to endocrinology specialists
**"Real-time audio + video intervention" (n=1)**						
**(Young et al. 2014)**<br><br>**US**	Sustained effects of a nurse coaching intervention via telehealth to improve health behavior change in diabetes	RCT	IG (n=51)<br>CG (n=50)	IG: Nurse Coaching Intervention via Telehealth (motivational interviewing technique; health behavior improvement); videoconferencing or telephone; once every 2 weeks<br>CG: usual care; not adequately described<br><br>9 months	• Significantly higher self-efficacy scores in IG compared with CG –based on the Diabetes Empowerment Scale at 9 months (4.03 versus 3.64, respectively; *P*<.05) and the difference in difference estimation (0.42; *P*<.05)<br>• Physical and mental health (HRQoL) improved, but no statistical significance (intergroup), *P*>.05	Positive:<br>Sustained effect on outcomes observed
**"Asynchronous interventions" (n=4)**						
**(Chen et al. 2013)**<br><br>**Taiwan**	Evaluating self-management behaviors of diabetic patients in a telehealth care program: longitudinal study over 18 months	RCT	IG (n=59)<br>CG (n=103)	IG: Online diabetes self-management system (data transmission and feedback via asynchronous text messages)<br>CG: not adequately described<br><br>18 months	• Five behaviors were significantly different between the intervention and control patients: being active (*P*<.001), healthy eating (*P*<.001), taking medication (*P*<.001), healthy coping (*P*=.02), and problem solving (*P*<.001)<br>• Significant reduction of HbA_{1c} level in intervention group (*P*=.02)	Positive
**(Istepanian et al. 2009)**<br><br>**UK**	Evaluation of a mobile phone telemonitoring system for glycaemic control	RCT	IG (n=72)<br>CG (n=65)	IG: mobile phone (bluetooth data transmission to web–based application for clinicians); feedback via letters<br>CG: usual care; contact as needed<br><br>9 months	• In a subgroup analysis of the patients who completed study, intervention group had lower HbA_{1c} than control group: 7.76% and 8.40%, respectively (*P*=.06)	Positive

Author, Year, Location	Topic	Study design	Participants/ included studies	Intervention and Control group	Main results	Overall value of intervention(s)
**(Fountoulakis et al. 2015)**<br><br>**Greece**	Impact and duration effect of telemonitoring on HbA_{1c}, BMI and cost in insulin-treated diabetes mellitus patients	RCT	IG (n=76)<br>CG (n=39)	IG: Telemonitoring system: data transmission via modem; feedback via mobile phone SMS or e-mail when needed<br>CG: usual care; not adequately described<br><br>6 months	• Significant reduction in HbA_{1c} in IG both at 3 [7.1±1.0% (54±10.5mmol/mol) *P*<.001] and 6 months [6.9±0.9% (52±9.5mmol/mol) *P*<.001], compared to CG<br>• Significant BMI reduction was observed in IG at 6 months on telemonitoring as well as at 6 months off telemonitoring compared to baseline<br>• Between-group analysis found no significant BMI alterations<br>• 6 months after telemonitoring, HbA_{1c} in IG was slightly increased [7.3±1.0%, 56±10.4 mol/mol]<br>• Compared to CG, number of monthly hypo- and hyperglycemias was reduced in IG<br>• Financial benefit for patients living more than 100km from the health care provider<br>• Cost of telemonitoring services per patient was 50 Euros bimonthly	Positive
**(Earle et al. 2010)**<br><br>**UK**	Mobile telemonitoring for achieving tighter targets of blood pressure control in patients with complicated diabetes	RCT, pilot	IG (n=72)<br>CG (n=65)	IG: mobile phone (bluetooth data transmission to web–based application for clinicians); feedback via letters<br>CG: usual care; contact as needed<br><br>9 months	• Systolic BP fell significantly in the patients in IG (mean [95% CI], -6.5 [-0.8 to -12.2] mmHg; *P*=.027) and remained unchanged in CG (2.1 [9.3 to -5.0] mmHg; *P*=.57)<br>• Those who achieved a systolic BP of <120mm Hg had lower average blood sugars than those with higher readings (7.8 [SD 1.6] vs. 8.9 [SD 2.2] mmol/L; *P*=.02)	Positive:<br>Mobile telemonitoring has potential
**"Combined interventions" (real-time and asynchronous communication) (n=2)**						
**(Leichter et al. 2013)**<br><br>**US**	Impact of remote management of diabetes via computer	RCT	IG (n=50)<br>CG (n=50)	IG: SMBG data transmission via internet; endocrinologist advice through e-mail and telephone<br>CG: quarterly clinic visits<br><br>12 months	• No significant between-group differences in HbA_{1c} (increase in IG), BP (increase of systolic BP in IG), lipids, or BMI (decrease in IG) were seen at 12 months<br>• IG subjects showed significantly greater reductions in mean (SD) body weight compared with CG subjects: - 5.2 (1.6) pounds versus - 0.7 (1.5) pounds (*P*=.04)<br>• Clinician time requirements for IG subjects were reduced by >40% (S N/R)	Mildly positive:<br>Feasible and efficient; similar clinical outcomes compared with traditional, clinic–based protocols

Author, Year, Location	Topic	Study design	Participants/ included studies	Intervention and Control group	Main results	Overall value of intervention(s)
**(Boaz et al. 2009)**<br><br>**Israel**	Automated telemedicine system improves patient-reported well-being	RCT	IG (n=17)<br>CG (n=18)	IG: telemedicine system: MedicGate (a receiver that transmits data to the information server; access to data via the internet); telephonic feedback by nurse<br>CG: were free to contact clinic personnel by telephone or visit clinic; scheduled to visit diabetes clinic every 3 months<br><br>6 months	• No significant differences in posttreatment metabolic parameters (IG: HbA_{1c} slight increase, fasting blood glucose decrease, lipids, weight slight increase), although serum glucose was marginally elevated in CG compared to IG (214 ± 65 mg/dL vs. 171 ± 77 mg/dL,*P*=.09)<br>• Being clinically symptom-free (71% vs. 11%, *P*=.003), having no hypoglycemic events (82% vs. 17%, *P*=.0001), and having no hyperglycemic events (65% vs. 17%, *P*=.004) were all significantly more frequently reported in IG (DRQoL measures)<br>• IG reported experiencing significantly less anxiety, treatment difficulty, depression, greater improvement in personal control over glucose, weight, and overall diabetes	Mildly positive:<br>Though posttreatment metabolic differences were not observed, IG reported significantly greater posttreatment experiences of improved QoL and sense of control

Abbreviations:

BP = blood pressure; CG = control group; DRQoL = diabetes-related quality of life; HbA_{1c} = glycated hemoglobin A_{1c}; HRQoL = health-related quality of life; IG = intervention group; MA = meta-analysis; MD = mean deviation; mHealth = mobile Health; N/S = Not significant; OR = odds ratio; QoL = quality of life; RCT = randomized controlled trial; SD = standard deviation; SMBG = self monitoring blood glucose; SMS = short message service; SR = systematic review; T1DM = type 1 diabetes mellitus; T2DM = type 2 diabetes mellitus
