# Supplementary material for: Clinical Improvements by Telemedicine Interventions Managing Type 1 and Type 2 Diabetes: Systematic Meta-review
Source: J Med Internet Res. 2021 Feb 19;23(2):e23244. doi: 10.2196/23244 (PMC7935656; doi:10.2196/23244)
Supplement: Multimedia Appendix 4 [file jmir_v23i2e23244_app4.pdf]

Quality assessments.

Quality appraisal using "A MeaSurement Tool to Assess systematic Reviews" (AMSTAR 2), n=21 studies

**Question/ Study**	PICO	Priori Design	Selec-tion study designs	Compre-hensive search strategy	Study selec-tion in dupli-cate	Data extrac-tion in dupli-cate	List of exclu-ded studies	Des-cription of studies	Assess-ment of risk of bias	Sorces of fun-ding	Statis-tical combi nation	Impact of risk of bias on results	Account for risk of bias when inter-preting results	Hetero-geneity	Publi-cation bias	Conflict of interest	**Overall assess-ment**
Faruque et al. 2017	+	+	+	+	+	+	-	+	+	+	+	+	+	+	+	+	**High**
Polisena et al. 2009	+	+	+	+	+	+	o	+	+	+	+	+	+	+	-	+	**High**
Su et al. 2016	+	+	+	+	+	+	o	+	+	-	+	+	+	+	+	+	**High**
Su et al. 2015	+	o	+	+	+	+	o	+	+	-	+	+	+	+	+	+	**High**
Tchero et al. 2019	+	o	+	+	+	+	+	+	+	-	+	+	+	+	+	+	**High**
Toma et al. 2014	+	o	+	+	+	+	o	+	+	-	+	+	+	+	+	+	**High**
Tao and Or 2013	+	o	+	+	+	+	o	+	+	-	+	+	+	+	+	+	**High**
Suksomboon et al. 2014	+	+	+	+	+	+	o	+	+	-	+	+	+	+	+	+	**High**
Hu et al. 2019	+	o	-	o	-	-	o	+	+	-	+	+	+	+	+	+	**Moderate**
Lee and Lee 2018	+	o	-	o	+	+	-	+	+	-	/	/	+	+	/	+	**Moderate**
Macdonald et al. 2017	+	o	-	o	+	+	-	+	+	-	/	/	+	+	/	*	**Moderate**
Marcolino et al. 2013	+	+	-	+	+	+	-	+	+	+	+	+	+	+	+	+	**Moderate**
So and Chung 2018	+	o	+	+	+	+	-	+	+	-	+	+	+	+	-	+	**Moderate**

**Question/ Study**	PICO	Priori Design	Selec-tion study designs	Compre-hensive search strategy	Study selec-tion in dupli-cate	Data extrac-tion in dupli-cate	List of exclu-ded studies	Des-cription of studies	Assess-ment of risk of bias	Sorces of fun-ding	Statis-tical combi nation	Impact of risk of bias on results	Account for risk of bias when inter-preting results	Hetero-geneity	Publi-cation bias	Conflict of interest	**Overall assess-ment**
Wu et al. 2018	+	o	+	+	+	+	o	+	+	-	+	+	+	+	-	+	**Moderate**
Hanlon et al. 2017	+	+	+	+	+	+	o	+	+	-	/	/	+	+	/	+	**Moderate**
Walker et al. 2017	+	+	+	+	-	-	o	+	+	-	/	/	+	+	/	+	**Moderate**
Baron et al. 2012	+	o	+	+	+	-	o	+	+	-	/	/	+	+	/	+	**Moderate**
Jong et al. 2014	+	o	+	+	-	-	o	+	+	-	/	/	+	+	/	+	**Moderate**
Kitsiou et al. 2017	+	o	+	+	+	+	o	+	+	+	/	/	+	+	/	+	**Moderate**
Teljeur et al. 2017	+	o	+	+	+	+	o	+	+	-	/	/	+	+	/	+	**Moderate**
Siriwardena et al. 2012	+	-	+	+	-	-	o	+	-	-	/	/	-	-	/	-	**Critically low**

+ = criterion met, - = criterion not met, o = criterion partially met, / = not applicable

Quality appraisal using “Effective Public Health Practice Project” (EPHPP), n=9 studies

**Question/ Study**	A Seletion Bias (Q1)	A Selection Bias (Q2)	A SCORE	B Study Design	B SCORE	C Confoun-ders (Q1)	C Confoun-ders (Q2)	C SCORE	D Blinding (Q1)	D Blinding (Q2)	D SCORE	E Data collection (Q1)	E Data Collection (Q2)	E SCORE	F Withdrawals and Drop-outs (Q1)	F Withdrawals and Drop-outs (Q2)	F SCORE	**GLOBAL RATING**
**“Real-time video interventions”**																		
Sood et al. 2018	1	1	***	1	***	1	1	**	2	3	**	1	1	***	3	4	*	**Moderate**
Kearns et al. 2012	4	5	*	2	***	2	/	***	3	3	**	1	1	***	3	4	*	**Weak**
**“Real-time video + audio interventions“**																		
Young et al. 2014	1	5	**	1	***	2	/	***	3	3	**	1	1	***	1	1	***	**Strong**
**“Asynchronous interventions“**																		
Istepanian et al. 2009	1	5	**	1	***	2	/	***	3	3	**	1	1	***	1	2	**	**Strong**
Fountoulakis et al. 2015	1	5	**	1	***	1	1	***	2	1	**	1	1	***	1	1	***	**Strong**
Earle et al. 2010	1	5	**	1	***	2	/	***	3	3	**	1	1	***	1	1	***	**Strong**
Chen et al. 2013	1	5	**	1	***	2	/	***	3	3	**	1	1	***	4	4	*	**Moderate**
**“Combined interventions“**																		
Leichter et al. 2013	1	1	***	1	***	2	/	***	3	3	**	1	1	***	1	2	**	**Strong**
Boaz et al. 2009	1	5	**	1	***	2	/	***	3	3	**	1	1	***	2	/	***	**Strong**

* = weak, ** = moderate, *** = strong, 1-5 = response options according to EPHPP, / = not applicable

Quality appraisal using "National Institute for Health and Care Excellence (NICE) for qualitative studies", n=1 study

**Question/ Study**	Is a qualitative approach appropriate ?	Is the study clear in what it seeks to do?	How defensible/ rigorous is the research design/ methodology?	How well was the data collectio carried out?	Is the role of the researcher adequately described?	Is the context clearly described?	Were the methods reliable?	Is the data analysis sufficiently rigorous?	Is the data "rich"?	Is the analysis reliable?	Are the findings convincing?	Are the finding relevant to the aims of the study?	Conclusions	How clear and coherent is the reporting of ethics?	**Overall assessment**
Fatehi et al. 2013<br>Real-time video intervention	Appropriate	Clear	Defensible	Appropriately	Unclear	Clear	Reliable	Rigorous	Ruch	Reliable	Convincing	Relevant	Adequate	Appropriate	**++**<br>(corresponds high)
