# Supplementary material for: Clinical Improvements by Telemedicine Interventions Managing Type 1 and Type 2 Diabetes: Systematic Meta-review
Source: J Med Internet Res. 2021 Feb 19;23(2):e23244. doi: 10.2196/23244 (PMC7935656; doi:10.2196/23244)
Supplement: Multimedia Appendix 5 [file jmir_v23i2e23244_app5.pdf]

Effects on primary outcome HbA_{1c} and secondary outcomes.

	Study	HbA_{1c}	FBG	BP	Body weight	BMI	DR QoL	HR QoL	Cost-effec-tiveness	Time saving	Other
Systematic Reviews & Meta-Analyzes	(Faruque et al. 2017)	+++					0	0			
	(Hu et al. 2019)	+++				(+++)					
	(Lee/Lee 2018)								+++		
	(Macdonald et al. 2017)										Enablers and Barriers using telemetry
	(Marcolino et al. 2013)	+++		(+++)^{sd}		(+++)					
	(Polisena et al. 2009)	+++ (S N/R)					+++ (S N/R)	+++ (S N/R)			
	(So/Chung 2018)	+++	(+++)								
	(Su et al. 2016)	+++ (smaller effect for T1DM)									
	(Su et al. 2015)	+++									
	(Tchero et al. 2019)	+++ (smaller effect for T1DM)							+++		
	(Toma et al. 2014)	+++ (smaller effect for T1DM)		+++^{sd}							
	(Wu et al. 2018)	+++		+++^{sd}		(+++)	(+++)	(+++)			
	(Hanlon et al. 2017)	+++ (only T2DM)									
	(Siriwardena et al. 2012)	+++							+++ (S N/R)		
	(Tao/Or 2013)	+++									
	(Walker et al. 2017)								+++ (S N/R)		
	(Baron et al. 2012)	+++ (S N/R)									
	(Jong et al. 2014)	+++ (S N/R)		+++ (S N/R)	+++ (S N/R)						
	(Kitsiou et al. 2017)	+++ (smaller effect for									

	Study	HbA_{1c}	FBG	BP	Body weight	BMI	DR QoL	HR QoL	Cost-effectiveness	Time saving	Other
"Real-time video interventions"		T1DM (not significant))									
	(Teljeur et al. 2017)								Not cost-effective		
	(Suksomboon et al. 2014)	(+++)									
	(Sood et al. 2018)	(++)		(- -)^{s}							
	(Kearns et al. 2012)	(+), (++)									
	(Fatehi et al. 2013)										Process/quality analysis of video consultations
"Real-time video & audio"	(Young et al. 2014)							(++)			
"Asynchronous interventions"	(Chen et al. 2013)	+									
	(Istepanian et al. 2009)	(++)									
	(Earle et al. 2010)			+^{s}							
	(Fountoulakis et al. 2015)	++ (not sustained)				+, (++)					
"Combined interventions"	(Leichter et al. 2013)	(- -)		(- -)^{s}	++	(++)				++ (S N/R)	
	(Boaz et al. 2009)	(- -)	(++)		(- -)		++				

Notes:
+++ = overall positive effects (SR/ MA)
++ = improvement in intervention group compared to control group (intergroup)
+ = improvement in intervention group compared to baseline (intragroup)

0 = no effect
- - = deterioration in intervention group compared to control group (intergroup)
- = deterioration in intervention group compared to baseline (intragroup)
( ) = not statistically significant

Abbreviations:
BP = blood pressure; d = diastolic blood pressure; DL = diabetes-related; FBG = fasting blood glucose; HbA_{1c} = glycated hemoglobin A_{1c}; HL = health-related; MA = meta-analysis; QoL = quality of life; RCT = randomized controlled trial; s = systolic blood pressure; S N/R = significance not reported; SR = systematic review; T1DM = type 2 diabetes mellitus; T2DM = type 2 diabetes mellitus
